# Machine learning-based diagnostic models for prevalent metabolic syndrome among patients with disuse muscle atrophy: a comparative study of seven algorithms

**DOI:** 10.3389/fendo.2026.1890881

**Published:** 2026-08-03

**Authors:** Jiaojiao Shu, Halimire Abudujilili, Wenhui Xiong, Lingyun Shi, Ailijiang Asila, Yujie Wang, Burebiguli Maimaiti, Gulizeba Wujimaimaiti, Palida Maimaiti

**Affiliations:** 1 School of Nursing, Xinjiang Medical University, Urumqi, China; 2 The First Affiliated Hospital of Xinjiang Medical University, Urumqi, China; 3 Traditional Chinese Medicine Hospital of Xinjiang Uyghur Autonomous Region, Urumqi, China; 4 Institute of Health Management, Xinjiang Medical University, Urumqi, China

**Keywords:** disuse muscle atrophy, machine learning, metabolic syndrome, retrospective survey, SHapley Additive exPlanations

## Abstract

**Background:**

Disuse muscle atrophy (DMA) leads to muscle loss and impaired motor function, while metabolic syndrome (MetS) increases the risk of cardiovascular disease and diabetes. Muscle loss exacerbates insulin resistance, accelerates MetS progression, and creates a vicious cycle. This study aimed to develop and validate an interpretable machine learning (ML) model for identifying prevalent MetS in patients with DMA.

**Methods:**

This retrospective study was conducted using data from 1,004 patients who visited the Department of Rehabilitation Medicine at four medical institutions in Urumqi, Xinjiang, between January 2018 and June 2024. The data were randomly divided into a training set (*n* = 704) and a validation set (*n* = 300). Feature selection was performed using least absolute shrinkage and selection operator (LASSO) regression with 10-fold cross-validation. Seven ML algorithms—Logistic Regression (LR), Decision Tree (DT), Random Forest (RF), XGBoost, LightGBM, Support Vector Machine (SVM), and Artificial Neural Network (ANN)—were used to construct diagnostic models. Model performance was evaluated using receiver operating characteristic curves, calibration plots, and decision curve analysis. The SHapley Additive exPlanations (SHAP) framework was applied to interpret the contributions of feature variables.

**Results:**

The prevalence of Mets among patients with DMA was 45.72%, eight key predictors were identified. The RF model demonstrated the best discriminatory performance, with an area under the curve (AUC) of 0.961 (95% CI: 0.938–0.979), accuracy of 90.0%, precision of 92.8%, sensitivity of 84.7%, specificity of 94.5%, and F1 score of 88.5%. The top five predictors identified by SHAP analysis were waist circumference, blood glucose, Cardiometabolic Index (CMI), High-density lipoprotein (HDL), and Triglyceride-Glucose index (TyG).

**Conclusion:**

We developed an interpretable ML model to aid in the early diagnosis of MetS. Among the models, particularly RF, showed outstanding predictive performance and promising application potential. It provides a practical tool for the early identification and targeted intervention, supports clinical decision-making, and promotes more rational allocation of healthcare resources.

## 1 Introduction

Disuse muscle atrophy (DMA) refers to the reduction in skeletal muscle protein synthesis and the increase in protein degradation caused by decreased limb activity, mechanical unloading, or immobilization due to various causes, leading to a decline in muscle mass, cross-sectional area, and strength (1, 2). It commonly occurs in conditions such as bed rest (e.g., intensive care unit−acquired weakness), external fixation after fracture, spinal cord injury, and a sedentary lifestyle in the elderly (3). DMA not only impairs limb motor function, increases the risk of falls and prolongs hospital stays, but also reduces basal metabolic rate, induces insulin resistance, and promotes fat deposition, thereby elevating the risk of developing multiple metabolic diseases (4–6).

Metabolic syndrome (MetS) is a clinical syndrome characterized mainly by abdominal obesity, hypertension, hyperglycemia, and dyslipidemia. It is a major risk factor for cardiovascular disease and type 2 diabetes, and its prevalence is increasing worldwide (7, 8). During periods of inactivity, muscle loss reduces the amount of insulin−responsive tissue, promoting insulin resistance and subsequently leading to metabolic dysfunction (9). This effectively accelerates the progression of MetS. In turn, metabolic dysfunction further contributes to muscle loss and disease progression, creating a vicious cycle (10).

Despite this situation, to our knowledge, no research team has yet developed a MetS diagnostic model specifically for patients with DMA. Existing tools are primarily designed for the general population or other high−risk groups and are not suitable for the unique pathophysiological conditions of DMA patients. Machine learning is an emerging technology for handling high-dimensional, nonlinear, and interactive clinical variables. It can extract key feature variables from large datasets for model development, thereby improving diagnostic accuracy and enabling precise interventions (11, 12). Although traditional machine learning outperforms conventional regression methods, its “black box” nature remains a major obstacle to clinical application (13). To improve the interpretability of machine learning models, researchers have employed the SHAP technique, which is based on a game–theory framework and significantly enhances the transparency of traditional models (14–16). This technique quantifies the contribution of each feature, helping us further identify the key predictive features associated with MetS in DMA patients and simplify the underlying models, thus facilitating the clinical translation of these findings.

Unlike prospective cohort studies that aim to predict long-term incidence of MetS, this study focuses on cross-sectional screening to detect the prevalence of MetS status at the time of hospital admission using routinely available clinical measurements. Therefore, the primary objectives of this study were: ① to develop seven distinct machine learning-based diagnostic models for screening prevalent MetS in patients with DMA using retrospective clinical data; ② to systematically and comprehensively compare the discrimination, calibration performance, and clinical utility of all models; and ③ to identify core predictive factors associated with MetS via SHAP-based interpretability analysis, thereby providing evidence for early clinical screening and targeted intervention in patients with DMA.

## 2 Methods

### 2.1 Data source

Data for the training and validation sets were obtained from the Department of Rehabilitation Medicine at four medical institutions in Urumqi, Xinjiang Uygur Autonomous Region, between January 2018 and June 2024. These retrospective data were used to build and validate the model. Prior to the study, the Institutional Review Board of the First Affiliated Hospital of Xinjiang Medical University approved the study protocol in accordance with the Declaration of Helsinki (Approval Number: 20200326-03). The four participating hospitals applied consistent diagnostic criteria for DMA and MetS, and their laboratory testing protocols complied with the Standard Operating Procedures for Clinical Laboratory Tests (WS/T 227-2024) issued by the National Health Commission of the People’s Republic of China, to minimize potential systematic measurement biases across institutions.

**Figure 1:**
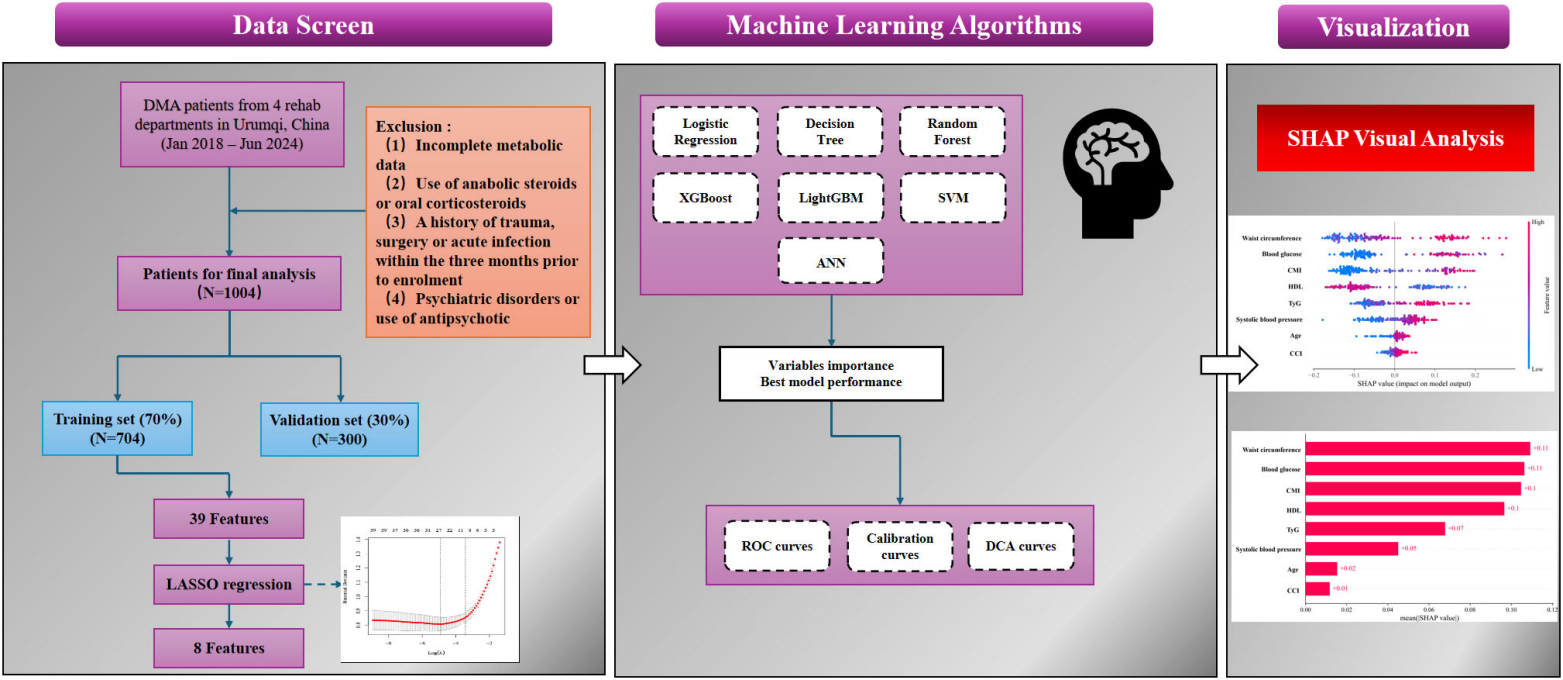
Study flow diagram.

This retrospective study was based on clinical data from participants diagnosed with DMA prior to enrollment. The inclusion criteria were as follows: ① meeting the diagnostic criteria for disuse muscular atrophy; ② Patients have been bedridden for ≥4 weeks due to the disease and having a Modified Barthel Index (MBI) score ≤60. Exclusion criteria included: ① incomplete metabolic parameter data in medical records; ② use of anabolic steroids or oral corticosteroids; ③ a history of trauma, surgery or acute infection within the three months prior to enrolment; ④ psychiatric disorders or use of antipsychotic medications. These criteria ensured the authenticity and reliability of the sample. A total of 1,004 cases were included in the final analysis. The participant screening process is detailed in **Figure 1**.

### 2.2 Basic information and specialized tests

Participants’ demographic data and specialized examination scores were obtained by reviewing the electronic medical record system. Demographic data included sex, age, marital status, educational level, occupation, use of mobility aids, method of medical cost coverage, place of residence, and history of pulmonary infection. Specialized examinations included the Charlson Comorbidity Index (CCI), the Functional Ambulation Classification (FAC), and the Medical Research Council (MRC) muscle strength score. All assessment metrics were based on results obtained on the day of admission. The specialized assessment tools are as follows:

#### 2.2.1 CCI

The participants were assessed using the CCI. The specific scoring criteria were as follows: myocardial infarction, congestive heart failure, peripheral vascular disease, cerebrovascular disease, dementia, chronic lung disease, connective tissue disease, peptic ulcer disease, mild liver disease, and diabetes were each assigned 1 point; hemiplegia, moderate or severe renal disease, diabetes with end−organ damage, any tumor without metastasis, leukemia, and lymphoma were assigned 2 points; moderate or severe liver disease was assigned 3 points; and Metastatic solid tumor and AIDS were assigned as high as 6 points. This assessment tool is primarily implemented based on patients’ clinical medical records and offers the advantages of being convenient, easily accessible, low-cost and highly clinically applicable (17).

#### 2.2.2 FAC

Gait function was assessed using the tool developed by Holden et al. (1984). This tool employs a six-level progressive scoring system: Level 0 indicates complete gait impairment (requiring a wheelchair or assistance from two or more people); Level 1 indicates crutch dependence (requiring continuous physical support from one person); Level 2 indicates assisted walking (requiring intermittent contact for support); Level 3 corresponds to supervised walking (requiring verbal guidance or behavioral supervision); Level 4 represents independent walking in a controlled environment (able to walk autonomously on flat ground but requiring supervision on complex terrain); and Level 5 signifies complete independence in walking function (18).

#### 2.2.3 MRC muscle strength rating

Muscle strength was evaluated using the grading system from the Medical Research Council (MRC). This scale defines six functional grades: Grade 0, no muscle contraction; Grade 1, flicker or trace of muscle contraction (visible or palpable contraction without joint movement); Grade 2, movement with gravity eliminated (joint movement possible but not against gravity); Grade 3, movement against gravity (movement possible against gravity but cannot resist resistance); Grade 4, movement against gravity and resistance (ability to withstand moderate resistance); and Grade 5, normal power against resistance (unrestricted movement in all directions). A total of 12 major muscle groups were evaluated, each scored from 0 to 5 based on functional performance, yielding a maximum total score of 60 points (19).

### 2.3 Laboratory test

The following data were obtained for the study participants by reviewing the electronic medical record system and laboratory reports: systolic blood pressure, diastolic blood pressure, waist circumference, white blood cell count, red blood cell count, hemoglobin, platelet count, Blood urea nitrogen, Serum creatinine, Serum uric acid, blood glucose, triglycerides, high-density lipoprotein (HDL), Serum calcium, total protein, albumin, globulin, aspartate aminotransferase (AST), alanine aminotransferase (ALT), Brain Natriuretic Peptid (BNP), interleukin-6 (IL-6), and D-dimer.

**Figure 2:**
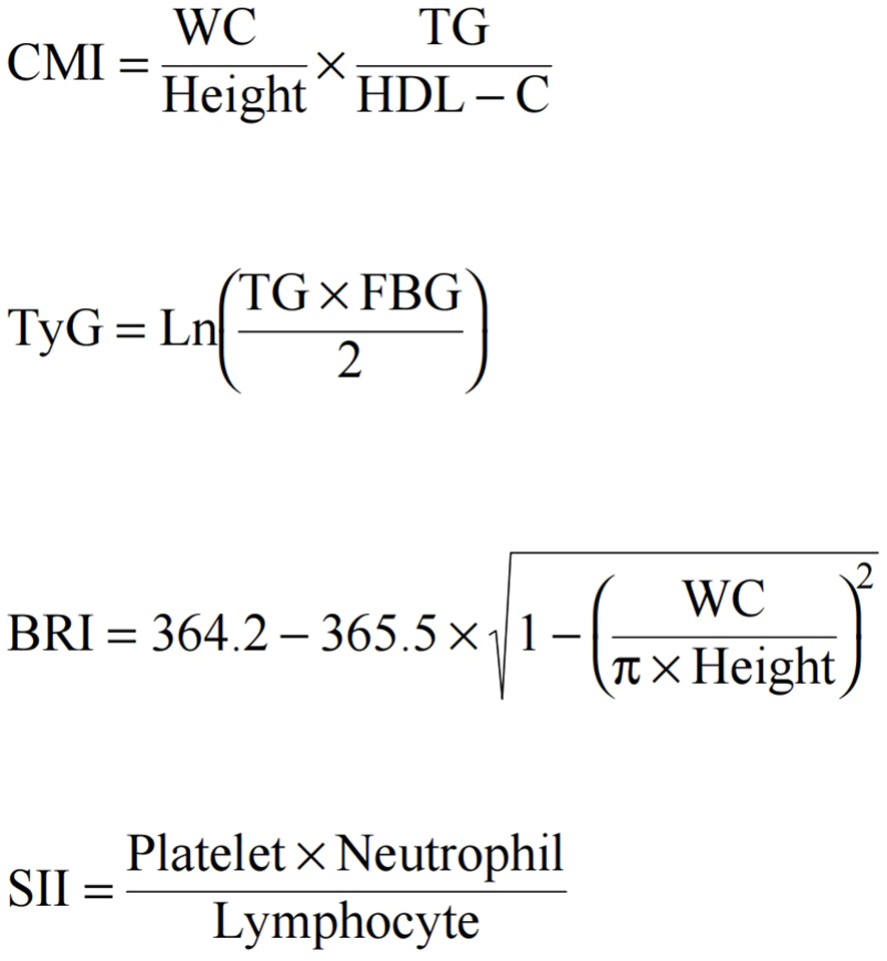
Chart of indicator formulas.

Based on these data, the following metabolic indices were calculated using relevant information from the electronic medical records: Cardiometabolic Index (CMI), Triglyceride-Glucose Index (TyG), Body Roundness Index (BRI), and Systemic Inflammation Index (SII) (20–23). The formulas for these metrics are shown in **Figure 2**.

### 2.4 Clinical outcomes

The primary outcome measure of this study was the occurrence of MetS. We used the diagnostic criteria for MetS established by the Diabetes Branch of the Chinese Medical Association, which were developed specifically for the Chinese population. A diagnosis of MetS was made when three or more of the following five criteria were met: (1) abdominal obesity: waist circumference ≥90 cm in men and ≥85 cm in women; (2) hypertension: systolic blood pressure ≥130 mmHg or diastolic blood pressure ≥85 mmHg or patients diagnosed with hypertension; (3) hyperglycemia: fasting blood glucose ≥6.1 mmol/L or blood glucose ≥ 7.8 mmol/L obtained 2 h after sugar loading or patients diagnosed with diabetes; (4) triglycerides ≥1.70 mmol/L; and (5) High-density lipoprotein cholesterol <1.04 mmol/L (24).

### 2.5 Data processing

Missing data are common in retrospective studies. To ensure an adequate sample size and minimize missingness, we calculated the missing rates for all candidate variables, as shown in **Table 1**. Accordingly, we used the multivariate imputation by chained equations algorithm implemented in the mice package in R. Variables with a missing rate exceeding 30% were excluded prior to imputation. The remaining variables with missing values were imputed using five complete datasets generated through iterative imputation. All analytical variables were included in the imputation models to ensure consistency. All imputation procedures were completed before data splitting to prevent information leakage between the training and testing sets (14).

**Table 1:** Missing values for each variable.

Variable	Missing ratio
Systolic blood pressure	0.009960159
Diastolic blood pressure	0.011952191
Waist circumference	0.006972112
CCI	0.006972112
CMI	0.015936255
TyG	0.009960159
SII	0.006972112
BRI	0.008964143
MRC muscle strength score	0.022908367
White blood cell count	0.010956175
Red blood cell count	0.001992032
Hemoglobin	0.002988048
Platelet count	0.008964143
Blood urea nitrogen	0.005976096
Serum creatinine	0.012948207
Serum uric acid	0.005976096
Blood glucose	0.025896414
Triglycerides	0.015936255
HDL	0.000996016
Serum calcium	0.005976096
Total protein	0.010956175
Albumin	0.001992032
Globulin	0.011952191
AST	0.001992032
ALT	0.000996016
BNP	0.011952191
IL-6	0.007968127
D-dimer	0.012948207
Age	0.000996016

### 2.6 Feature selection

Feature selection was performed using the Least Absolute Shrinkage and Selection Operator (LASSO), which selects variables by applying an L1 regularization penalty to the regression coefficients. LASSO analysis typically resolves multicollinearity among predictors, thereby improving model stability (25, 26). The optimal regularization parameter (λ) was determined using 10−fold cross−validation. Notably, all steps were conducted on the training set. The selected variables were then fixed and directly applied to the validation set without further screening, ensuring model interpretability.

### 2.7 Model development and evaluation

This study employed seven machine learning algorithms to build diagnostic models, including logistic regression (LR), decision trees (DT), random forests (RF), extreme gradient boosting (XGBoost), light gradient boosting machine (LightGBM), support vector machines (SVM), and artificial neural networks (ANN). All models were implemented in Python 3.12.4. The core libraries used included pandas, NumPy, stats models, scikit-learn, XGBoost, LightGBM, SHAP, matplotlib, seaborn, and SciPy. A random seed of 123 was used for all stochastic processes, including dataset splitting, cross-validation, and hyperparameter tuning.

Before data integration, all patient identifiers (including the treating hospital) were removed in accordance with the research management department’s requirements to protect privacy. Consequently, center−based stratification was not possible. Therefore, we randomly split the patients into a training set (70%) and an internal validation set (30%) using simple random sampling. This study included a total of 1,004 participants, of whom 459 had MetS and 545 did not. The sample was well balanced, with no significant class imbalance. Therefore, neither resampling techniques nor sample weight adjustments were applied during model training.

During hyperparameter optimization, five−fold cross−validation was used as the internal splitting scheme for the training set. For the six models—DT, RF, XGBoost, LightGBM, SVM, and ANN—grid search was performed with the area under the receiver operating characteristic (ROC) curve (AUC) as the evaluation metric to identify the optimal parameter combinations. Since LR is a linear model, it does not require hyperparameter tuning; we simply incorporated the selected features and added a constant term to complete the fit. The complete parameter search ranges and optimal settings for each model are detailed in **Table 2**.

**Table 2:** Parameter search ranges and optimal parameter settings for each model.

Model	Hyperparameter search range	Final optimized parameters
LR	No hyperparameter tuning required	Intercept included all LASSO-screened predictors input
DT	max_depth: [3,5,10,None]; min_samples_split: [5,10,20]; max_features: [‘sqrt’,None]; ccp_alpha: [0.0,0.01,0.1]	ccp_alpha=0.0, max_depth=5, max_features=None, min_samples_split=20
RF	n_estimators: 50–450 (step 50); max_features: 2 to sqrt(total features)	n_estimators=300, max_features=3
XGBoost	learning_rate: [0.01,0.05,0.1]; max_depth: [3,5,7]; n_estimators: [50,100,150]; subsample: [0.6,0.8,1.0]; reg_alpha: [0,0.1,0.5]; reg_lambda: [1,1.5,2.0]	learning_rate=0.05, max_depth=7, n_estimators=100, reg_alpha=0, reg_lambda=1.5, subsample=0.6
LightGBM	learning_rate: [0.1,0.2]; num_leaves: [31,50,100]; n_estimators: [100]; subsample: [0.6,0.8,1.0]; colsample_bytree: [0.6,0.8,1.0]	colsample_bytree=0.6, learning_rate=0.2, n_estimators=100, num_leaves=31, subsample=0.6
SVM	C: [0.1]; kernel: [‘linear’,’rbf’]; gamma: [‘scale’,’auto’,0.01]; degree: [2,3,4]	C=0.1, degree=2, gamma=‘scale’, kernel=‘linear’
ANN	hidden_layer_sizes: [(25),(50),(100),(10,10),(50,50),(100,100),(50,50,50)]; activation: [‘relu’,’tanh’,’logistic’]	activation=‘logistic’, hidden_layer_sizes=(50,50)

Model performance evaluation includes: ① assessing discriminatory ability using metrics such as ROC-AUC, accuracy, sensitivity, specificity, precision, and F1 score; ② evaluating calibration using calibration curves and the Brier score; and ③ assessing clinical utility through decision curve analysis (DCA). Together, these metrics provide a comprehensive assessment of the model’s diagnostic performance.

### 2.8 Assessment of variable importance

To assess variable importance, the SHAP method—a game-theory-based approach for interpreting machine learning results—was employed (27–29). This method enhances model interpretability and effectively addresses the “black box” limitation inherent in traditional algorithms. SHAP values were calculated to quantify the contribution of each variable to the model’s predictions. Bar charts, bee swarm plots, and dependency plots were used to provide an overall overview, while waterfall plots and force plots offered more intuitive individual explanations. In addition, to enhance clinical practicality, we constructed nomograms for these eight feature variables.

### 2.9 Statistical analysis

Continuous variables were expressed as mean ± standard deviation (SD), and categorical variables as frequency (n) and percentage (%). Between-group comparisons for normally distributed variables were performed using the independent samples t−test; for non−normally distributed variables, the Mann−Whitney U test was used. Categorical variables were analyzed using the chi−square test. A two−sided P−value < 0.05 was considered statistically significant. All statistical analyses were performed using R version 4.6.0 (R Statistical Computing Foundation) and Python version 3.12.4 (Python Software Foundation).

## 3 Results

### 3.1 Subject characteristics

After applying the exclusion criteria, 1,004 participants were included in the development of the machine learning diagnostic model. Among them, 459 (45.7%) were diagnosed with MetS, and 545 (54.3%) were not. Across the entire study population, 63.35% were female, 90.74% were married, 42.33% had an education level of Junior high school and below, and 73.31% resided in urban areas.

Significant differences between the MetS and non-MetS groups were observed for multiple variables, including sex, marital status, occupation type, age, systolic and diastolic blood pressure, waist circumference, CCI, CMI, TyG, BRI, white blood cell count, hemoglobin, blood urea, serum creatinine, serum uric acid, blood glucose, triglycerides, HDL, total protein, globulin, AST, ALT, and BNP (all *P* < 0.05). In contrast, no significant differences were found in education level, use of walking aids, medical payment method, Residence, Pulmonary infection, FAC, MRC muscle strength score, SII, Red blood cell count, platelet count, serum calcium, albumin, IL-6, and D-dimer (*P* > 0.05). Detailed information is provided in **Table 3**.

**Table 3:** Baseline characteristics table of the present study (data presented as frequency (percentage) or mean ± standard deviation (SD) or median [(IQR)].

Variables	Total (n = 1004)	DMA without MetS (n = 545)	DMA with MetS (n = 459)	P
Gender, n (%)				0.001
Male	368 (36.65)	225 (41.28)	143 (31.15)	
Female	636 (63.35)	320 (58.72)	316 (68.85)	
Marital status, n (%)				0.003
Married	911 (90.74)	480 (88.07)	431 (93.90)	
Unmarried	60 (5.98)	45 (8.26)	15 (3.27)	
Other	33 (3.29)	20 (3.67)	13 (2.83)	
Education level, n (%)				0.141
Junior high school and below	425 (42.33)	235 (43.12)	190 (41.39)	
Senior high school	183 (18.23)	110 (20.18)	73 (15.90)	
Junior college	299 (29.78)	148 (27.16)	151 (32.90)	
Bachelor’s degree and above	97 (9.66)	52 (9.54)	45 (9.80)	
Occupation, n (%)				0.019
Staff	198 (19.72)	106 (19.45)	92 (20.04)	
Worker	38 (3.78)	29 (5.32)	9 (1.96)	
Individual	140 (13.94)	68 (12.48)	72 (15.69)	
Farmer	130 (12.95)	69 (12.66)	61 (13.29)	
Retirement	397 (39.54)	210 (38.53)	187 (40.74)	
Unemployed	50 (4.98)	27 (4.95)	23 (5.01)	
Other	51 (5.08)	36 (6.61)	15 (3.27)	
Use of walking aids, n (%)				0.152
No	225 (22.41)	122 (22.39)	103 (22.44)	
Require assisted walking or wall support	40 (3.98)	20 (3.67)	20 (4.36)	
Wheelchair assistance	292 (29.08)	174 (31.93)	118 (25.71)	
Completely bedridden	447 (44.52)	229 (42.02)	218 (47.49)	
Medical payment method, n (%)				0.447
Medical Insurance	951 (94.72)	513 (94.13)	438 (95.42)	
New rural cooperative medical scheme	34 (3.39)	19 (3.49)	15 (3.27)	
Self-funded	19 (1.89)	13 (2.39)	6 (1.31)	
Residence, n (%)				0.718
Urban	736 (73.31)	397 (72.84)	339 (73.86)	
Rural	268 (26.69)	148 (27.16)	120 (26.14)	
Pulmonary infection, n (%)				0.062
Yes	609 (60.66)	345 (63.30)	264 (57.52)	
No	395 (39.34)	200 (36.70)	195 (42.48)	
Functional ambulation classification, n (%)				0.120
Level 0	686 (68.33)	353 (64.77)	333 (72.55)	
Level 1	89 (8.86)	52 (9.54)	37 (8.06)	
Level 2	152 (15.14)	94 (17.25)	58 (12.64)	
Level 3	69 (6.87)	42 (7.71)	27 (5.88)	
Level 4	7 (0.70)	4 (0.73)	3 (0.65)	
Level 5	1 (0.10)	0 (0.00)	1 (0.22)	
Age, years (median, IQR)	57.00 (47.00, 67.00)	57.00 (42.50, 66.00)	58.00 (50.00, 68.00)	0.003
MRC muscle strength score, points (median, IQR)	42.00 (36.00, 54.00)	42.00 (36.00, 54.00)	43.00 (36.00, 53.00)	0.865
Systolic blood pressure, mmHg (median, IQR)	130.00 (120.00, 145.00)	125.00 (116.00, 140.00)	135.00 (125.00, 150.00)	<0.001
Diastolic blood pressure, mmHg (median, IQR)	80.00 (73.00, 88.00)	79.00 (72.00, 86.00)	81.00 (75.00, 90.00)	<0.001
Waist circumference, cm (median, IQR)	86.80 (80.00, 93.00)	83.00 (77.60, 88.00)	91.60 (85.00, 96.50)	<0.001
CCI, points (median, IQR)	3.00 (2.00, 4.00)	3.00 (2.00, 4.00)	3.00 (2.00, 5.00)	<0.001
CMI, (median, IQR)	0.62 (0.42, 1.02)	0.47 (0.35, 0.64)	0.97 (0.66, 1.47)	<0.001
TyG, (median, IQR)	8.68 (8.30, 9.09)	8.46 (8.13, 8.74)	9.04 (8.67, 9.46)	<0.001
SII, (median, IQR)	701.80(407.95, 1362.47)	700.88 (398.63, 1296.64)	712.12 (419.33, 1412.73)	0.528
BRI, (median, IQR)	30.08 (28.09, 32.36)	29.01 (27.46, 30.79)	31.76 (29.42, 33.70)	<0.001
White blood cell count, 10^9^/L (median, IQR)	7.44 (5.73, 10.20)	7.26 (5.51, 9.52)	7.80 (5.99, 10.84)	<0.001
Red blood cell count, 10^12^/L (median, IQR)	4.19(3.65,4.70)	4.15 (3.65, 4.65)	4.24 (3.66, 4.75)	0.05
Hemoglobin, g/L (mean ± SD)	123.64 ± 21.43	122.14 ± 20.42	125.42 ± 22.47	0.016
Platelet count, 10^9^/L (median, IQR)	239.00 (187.25, 305.00)	238.00 (187.50, 313.00)	239.00 (187.00, 296.00)	0.689
Blood urea nitrogen, mmol/L (median, IQR)	5.18 (4.16, 6.45)	5.00 (4.01, 6.16)	5.41 (4.32, 7.00)	<0.001
Serum creatinine, μmol/L (median, IQR)	57.70 (47.64, 73.40)	56.09 (45.89, 67.50)	63.54 (50.40, 76.50)	<0.001
Serum uric acid, μmol/L (median, IQR)	267.95 (196.06, 336.95)	258.76 (184.44, 320.75)	275.01 (211.71, 356.76)	<0.001
Blood glucose, mmol/L (median, IQR)	5.71 (5.04, 7.18)	5.32 (4.80, 5.93)	6.72 (5.47, 8.31)	<0.001
Triglycerides, mmol/L (median, IQR)	1.26 (0.90, 1.72)	1.08 (0.81, 1.37)	1.60 (1.13, 2.15)	<0.001
HDL, mmol/L (median, IQR)	1.04 (0.81, 1.25)	1.15 (0.99, 1.33)	0.87 (0.71, 1.03)	<0.001
Serum calcium, mmol/L (median, IQR)	2.20 (2.12, 2.29)	2.20 (2.11, 2.28)	2.20 (2.12, 2.29)	0.687
Total protein, g/L (median, IQR)	65.00 (60.45, 70.32)	64.45 (60.20, 69.58)	65.70 (61.01, 71.90)	0.010
Albumin, g/L (median, IQR)	36.01(32.10,39.48)	35.76(32.45, 38.97)	36.30(31.82,40.20)	0.221
Globulin, g/L (mean ± SD)	29.83 ± 5.62	29.32 ± 5.58	30.43 ± 5.61	0.002
AST, U/L (median, IQR)	27.00 (20.69, 38.17)	26.36 (20.00, 37.98)	28.00 (21.40, 38.50)	0.045
ALT, U/L (median, IQR)	25.00 (16.38, 41.00)	23.00 (15.34, 41.00)	26.25 (17.81, 41.00)	0.004
BNP, pg/mL (median, IQR)	102.12 (54.33, 280.25)	97.36 (53.11, 216.00)	114.00 (55.70, 402.00)	0.013
IL-6, pg/L (median, IQR)	10.28 (4.86, 26.53)	9.49 (4.64, 27.15)	10.86 (5.07, 25.90)	0.338
D-dimer, ng/mL (median, IQR)	231.00 (107.00, 565.50)	240.00 (115.00, 565.00)	218.00 (101.00, 566.00)	0.523

### 3.2 Feature selection

**Figure 3:**
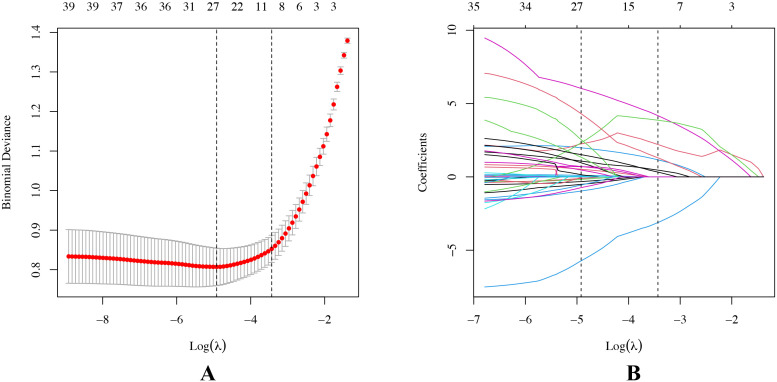
Results of LASSO regression analysis. **(A)** Coefficient trajectory of all 39 variables under different regularization parameters. **(B)** 10-fold cross-validation curve. The left vertical dashed line represents the minimum λ, and the right vertical dashed line corresponds to λ within one standard error.

In this study, LASSO regression was used for variable selection, and the model was tuned using 10-fold cross-validation; the results are shown in **Figure 3**. In the LASSO regression analysis, the minimum value of λ and the value of λ plus one standard error were 0.007 and 0.032, respectively. To avoid the risk of overfitting, we selected the value of λ plus one standard error as the optimal candidate value. Based on this analysis, eight feature variables were ultimately retained: systolic blood pressure, waist circumference, CCI, CMI, blood glucose, TyG, HDL, and age. These variables will serve as input data for subsequent machine learning model development.

### 3.3 Model performance evaluation

**Figure 4:**
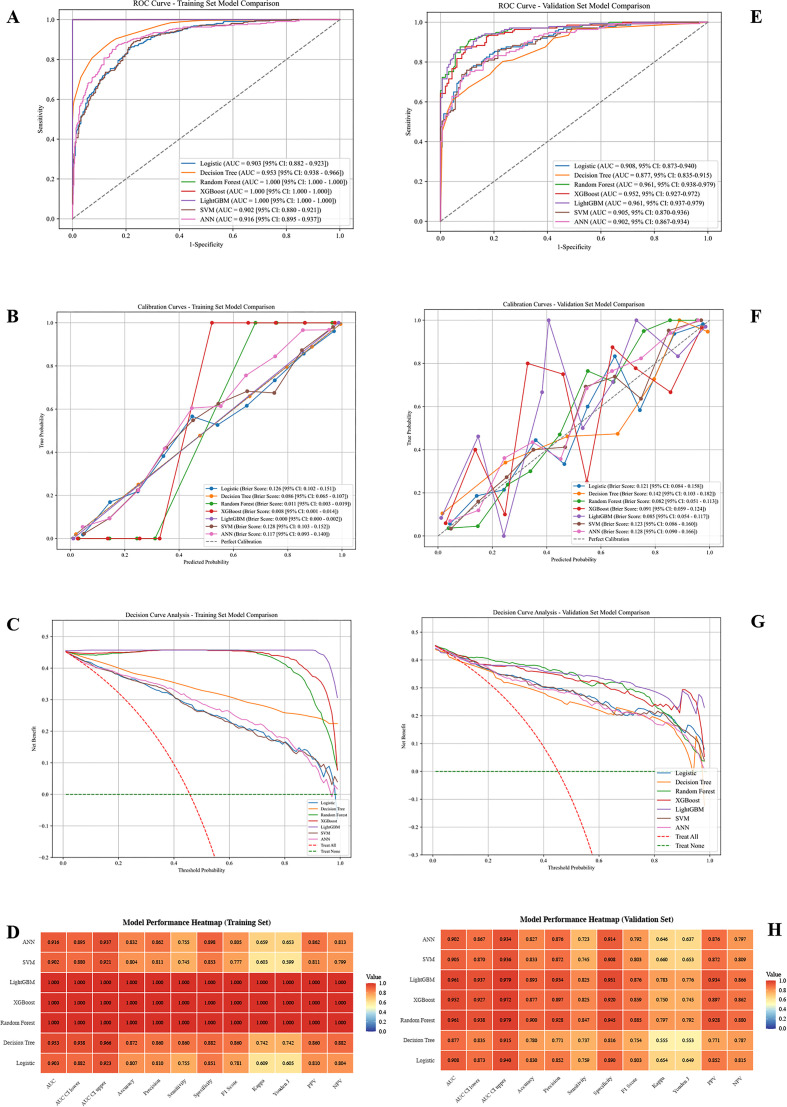
**(A)** shows the ROC curves, **(B)** displays the calibration curves, and **(C)** presents the DCA curves of seven machine learning models in the training set; **(D)** is the correlation heatmap for performance comparison of the seven machine learning models in the training set. In addition, **(E–G)** separately illustrate the ROC curves, calibration curves and DCA curves of seven machine learning models in the validation set, and **(H)** represents the correlation heatmap for performance comparison of the seven machine learning models in the validation set.

To diagnose MetS in patients with DMA, we developed seven machine learning models and evaluated them on the training and validation sets. The performance of these models is shown in **Figure 4**, illustrated by ROC curves, calibration curves, DCA curves, and correlation heatmaps. In the validation set, the RF model performed more robustly than the other models (AUC: 0.961, 95% CI: 0.938–0.979; accuracy: 0.900; precision: 0.928; sensitivity: 0.847; specificity: 0.945; F1 score: 0.885).

The calibration curves for the training and validation sets showed that the RF model’s calibration curve most closely approximated the ideal curve. The Brier score was 0.011 (95% CI: 0.003–0.019) for the training set and 0.082 (95% CI: 0.051–0.113) for the validation set (see legends of **Figures 4B, F**). Although the RF model performed slightly worse than LR in terms of calibration intercept and slope, it still yielded the most favorable overall results, with a maximum net benefit of 0.452 and an effective threshold range of 0.01 to 0.99. Detailed results are presented in **Table 4** and **Table 5**.

**Table 4:** Calibration intercepts and calibration slopes for each model.

Model	Calibration slope	Calibration intercept
LR	1.0342	0.0065
DT	0.8138	0.1207
RF	1.1767	-0.0635
XGBoost	0.9805	0.0433
LightGBM	0.6496	0.2733
SVM	1.0585	0.0038
ANN	1.1436	-0.121

**Table 5:** Maximum net benefit values and valid risk threshold ranges for each model.

Model	Maximum net benefit	Valid risk threshold range
LR	0.452	[0.01, 0.99]
DT	0.378	[0.13, 0.93]
RF	0.452	[0.01, 0.99]
XGBoost	0.449	[0.02, 0.99]
LightGBM	0.413	[0.08, 0.98]
SVM	0.451	[0.01, 0.99]
ANN	0.451	[0.01, 0.98]

**Figure 5:**
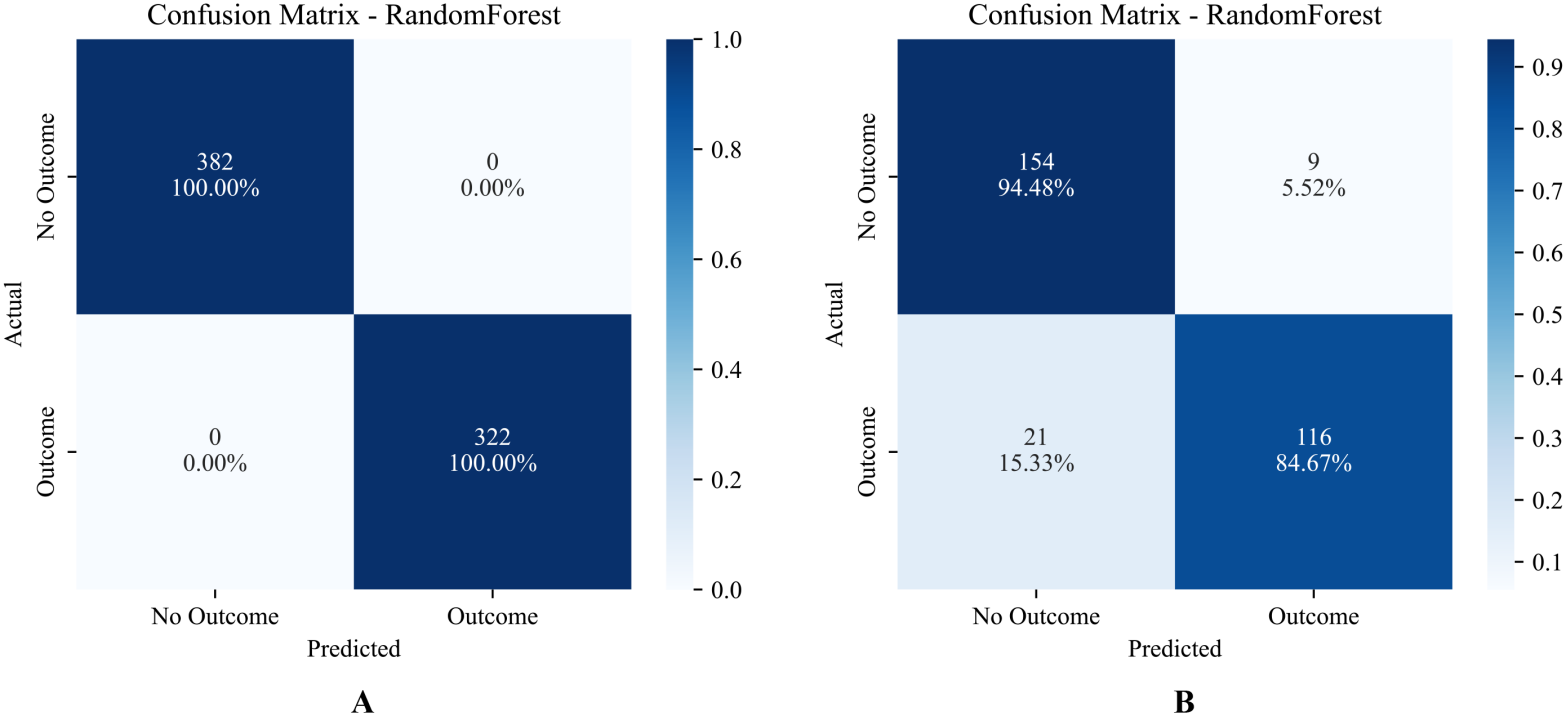
**(A)** and **(B)** represent the confusion matrix plots of the RF model in the training set and validation set, respectively.

Although the LightGBM model’s AUC value on the validation set was nearly identical to that of the RF model (0.961, 95% CI: 0.937–0.979), its sensitivity (0.825) was slightly lower than that of the RF model(0.847), and its calibration curve performed poorly. The XGBoost model also performed well on the validation set (AUC: 0.952, sensitivity: 0.825), but it was slightly inferior to the RF model in terms of calibration and clinical net benefit. In addition, the confusion matrices for the training and validation sets further demonstrated the classification performance of the RF model. On the validation set, the random forest model correctly identified 154 of 163 negative cases (specificity: 94.48%) and 116 of 137 positive cases (sensitivity: 84.67%), resulting in 9 false positives and 21 false negatives. Detailed results are shown in **Figure 5**.

To evaluate the reliability of the optimal RF model, we performed 1,000 bootstrap resampling iterations for internal validation to quantify optimism bias. The RF model achieved an apparent AUC of 0.958 on the development dataset, with a bootstrap−estimated optimism of 0.046, yielding a bias−corrected AUC of 0.912. On the independent test set, the model attained a raw AUC of 0.961. These results suggest slight overfitting; nevertheless, the model’s discriminative performance remains reliable. The calibration slope and intercept indicated only minor bias, further confirming good model reliability.

#### 3.3.1 Sensitivity analysis of feature input space

To assess whether the variable selection strategy affects model performance, we conducted a sensitivity analysis. Seven machine learning models were developed using all candidate variables without LASSO filtering, and their validation set AUCs were compared with those of models built using LASSO−selected variables. Detailed results are presented in **Table 6**. The results showed that the AUCs of models using all candidate variables ranged from 0.724 to 0.955. After dimensionality reduction via LASSO regression, the AUCs for LR, SVM, and ANN improved substantially; those for RF, LightGBM, and XGBoost remained largely unchanged compared with the full−variable models; and only the AUC for DT decreased slightly. These findings confirm that LASSO effectively eliminates irrelevant variables, reduces data noise, and enhances model discriminative ability.

**Table 6:** Comparison of AUC values on the validation set between models built using all candidate variables and models built using variables selected via LASSO.

Model	AUC (all candidate variables)	AUC (8 LASSO-screened predictors)
LR	0.880	0.908
DT	0.916	0.877
RF	0.946	0.961
XGBoost	0.955	0.952
LightGBM	0.955	0.961
SVM	0.877	0.905
ANN	0.724	0.902

### 3.4 Model interpretation

**Figure 6:**
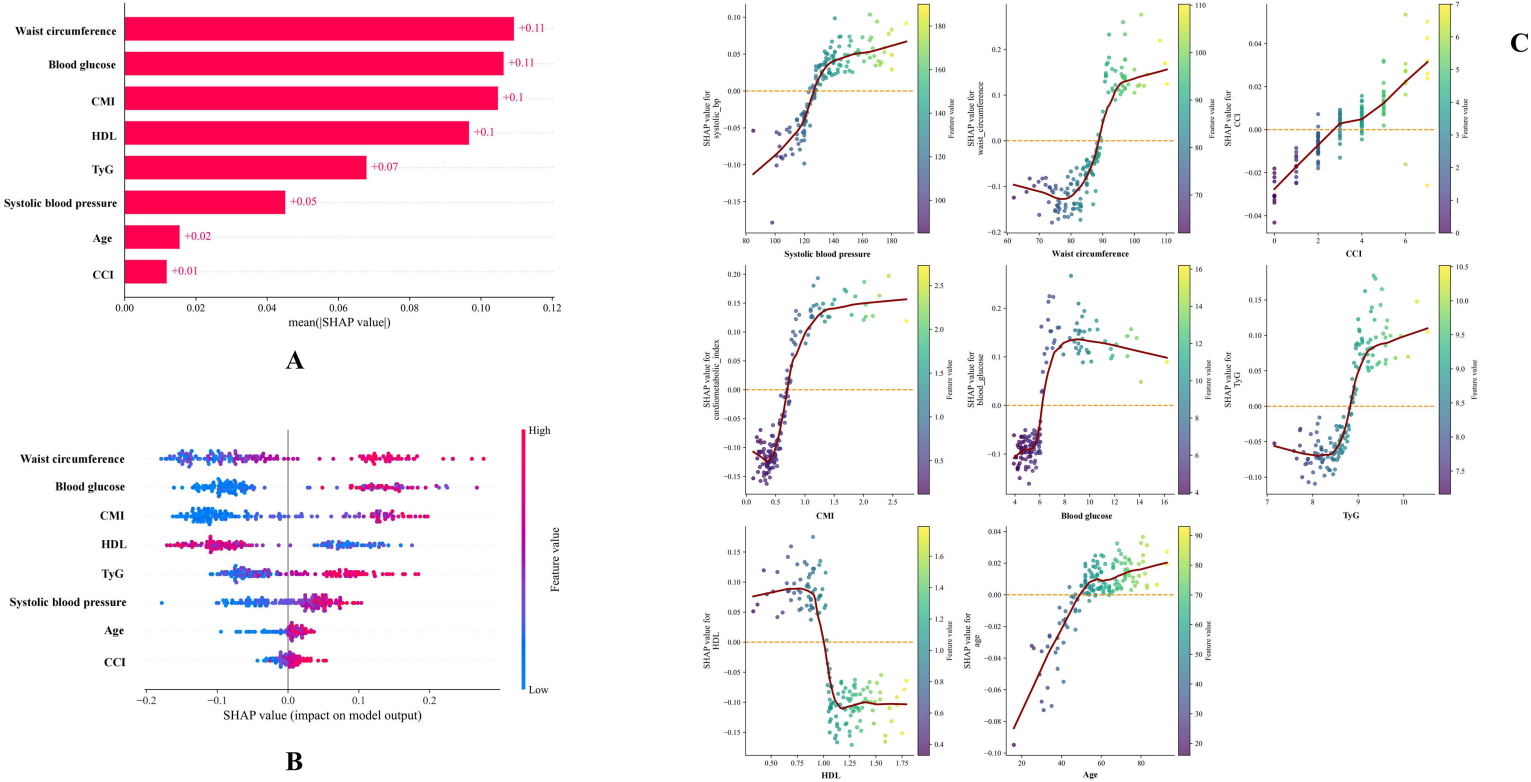
Feature importance analysis of MetS in DMA patients identified by the RF model. **(A)** illustrates the ranking of feature variables based on mean absolute SHAP values, with feature importance decreasing from top to bottom. **(B)** displays variables ranked by their contribution to MetS classification in descending order, and the vertical axis is set at a Shap value of 0. Red dots on the right of the axis stand for positive contributions indicating elevated MetS risk, whereas blue dots on the left represent negative contributions indicating reduced MetS risk. **(C)** The dependence plots intuitively visualize the influences of these eight features on the model.

To gain further insight into the decision-making process of the RF model, we performed SHAP analysis to quantify the contribution and effects of each feature on the model’s predictions; the results are shown in **Figure 6**.

**Figure 6A** presents the ranking of feature variables based on the mean absolute SHAP value, with feature importance decreasing from top to bottom. **Figure 6B** displays the variables sorted from top to bottom by their contribution to MetS classification, with the vertical axis defined at a SHAP value of 0. Red points to the right of the axis indicate a positive contribution (i.e., increased MetS risk), whereas blue points to the left of the axis indicate a negative contribution (i.e., decreased MetS risk). **Figure 6C** serves as a dependency plot, providing a more intuitive visualization of the impact of these eight features on the RF model. The results indicate that increased waist circumference, blood glucose, CMI, TyG, systolic blood pressure, age, and CCI are positively associated with the development of MetS. Conversely, higher HDL levels are negatively associated with the development of MetS.

### 3.5 Clinical applications of the model

**Figure 7:**
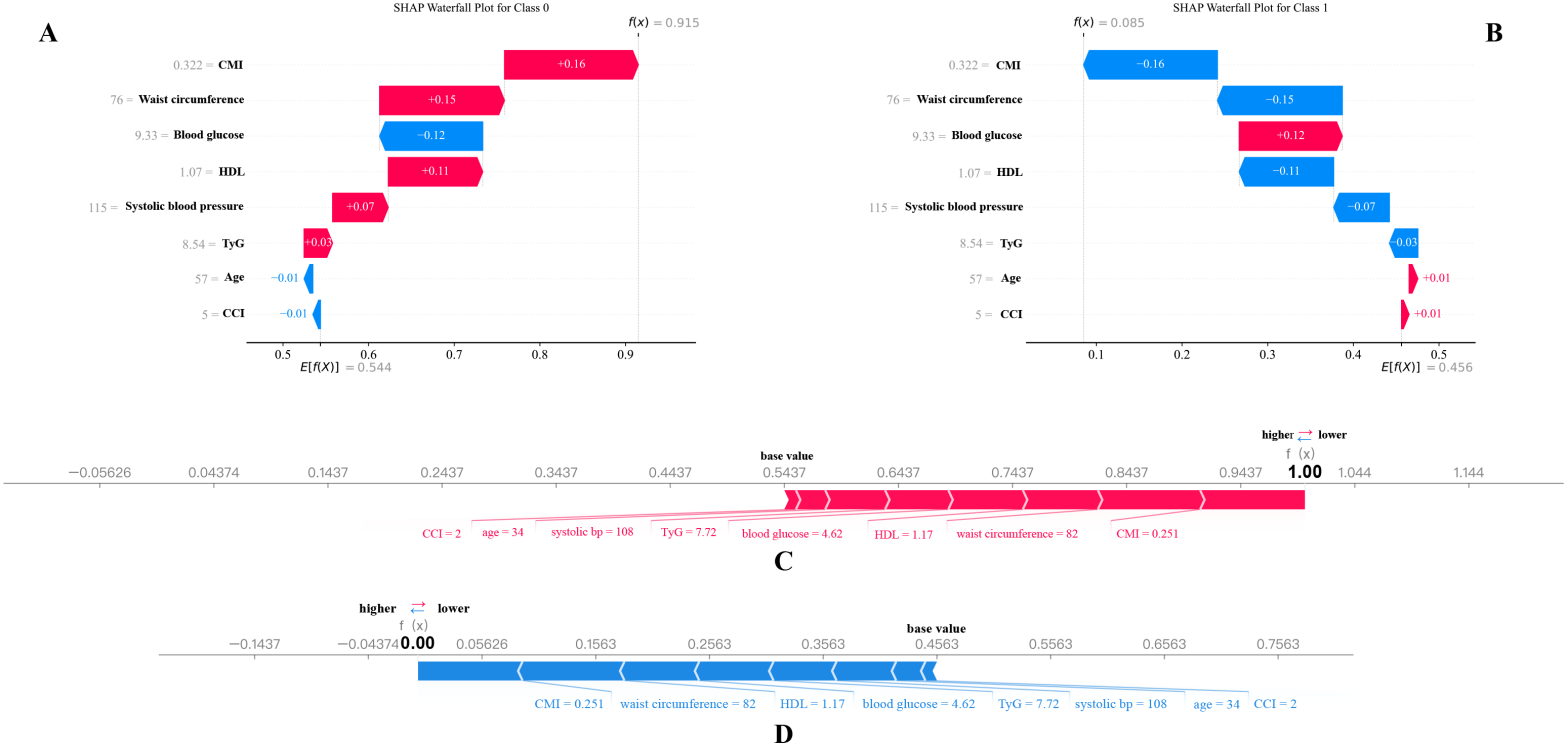
Visualization diagrams of individual classifications derived from SHAP interpretable analysis. **(A, B)** are waterfall plots that intuitively present the cumulative contribution of each variable from baseline value to the final output in the samples. **(C, D)** are force plots demonstrating the contribution decomposition of each feature.

To demonstrate how the RF model evaluates the contribution of each patient characteristic, two patients were randomly selected to evaluate the contribution of each characteristic to the model’s classification of MetS (vs. non-MetS). The results are shown in **Figure 7**. The waterfall plots and force plots illustrate the specific contributions of these characteristics to the RF model’s classification of MetS, which are consistent with the findings from the dependency and swarm plots.

### 3.6 Construction of a nomogram

**Figure 8:**
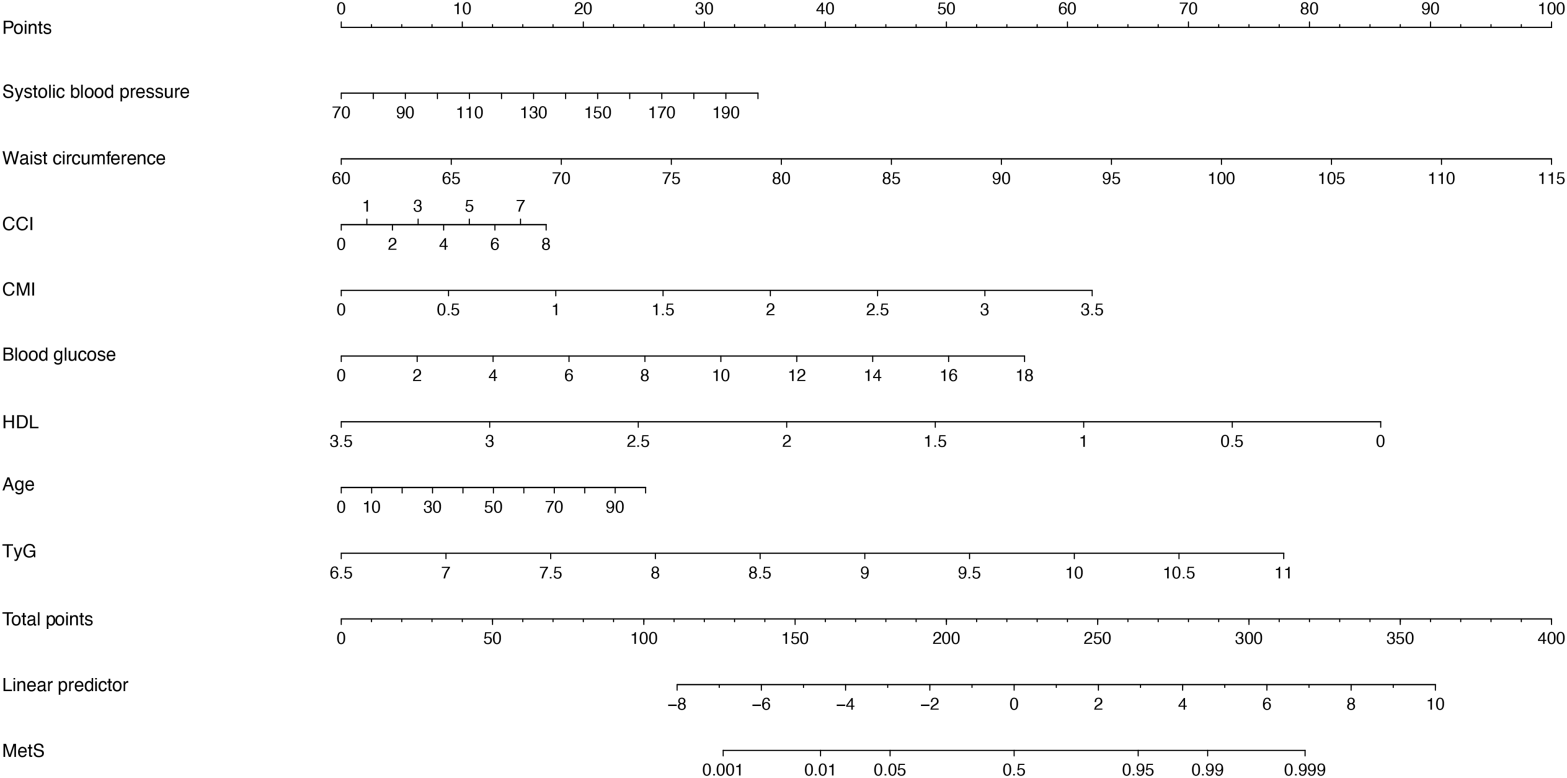
The Nomogram for the LR Model.

To facilitate the clinical application of this model, we constructed a nomogram based on the eight feature variables identified above. As shown in **Figure 8**, each variable corresponds to an independent point score. After summing these scores to obtain a total score, the linear predictor and the corresponding probability of having MetS can be derived using the risk axis at the bottom of the chart.

## 4 Discussion

To our knowledge, this is the first study to use machine learning algorithms to diagnose prevalent MetS in patients with DMA. Using retrospectively collected clinical data and various machine learning algorithms, we identified key predictive features correlated with MetS in DMA patients. Our diagnostic model can screen for existing MetS, support clinical decision-making, and thus holds significant clinical value.

Due to muscle loss, patients with DMA often develop sarcopenic obesity, which increases their risk of MetS (30–32). This situation underscores the importance of early and accurate identification. To address this clinical need, this study developed seven machine learning models. Through rigorous and systematic evaluation, the RF model was identified as the optimal clinical model. It not only achieved a high AUC but also performed well in calibration curve and DCA. In clinical diagnosis, an AUC value exceeding 0.7 indicates that a model can effectively distinguish between affected and unaffected individuals. Therefore, the RF model developed in this study can be regarded as a practical clinical decision−making tool to serve clinicians and patients.

This study has one inherent limitation: some of the feature variables included partially overlap with the diagnostic criteria for MetS. To some extent, this may have artificially inflated the AUC of all models, raising potential concerns regarding circularity and data leakage. Nevertheless, these variables are routinely collected during admission examinations for DMA patients and are readily accessible in clinical practice, allowing the model to perform preliminary screening before a formal MetS diagnosis, thus demonstrating practical utility.

To confirm that the model’s strong discriminatory ability was not solely attributable to this overlap, we performed sensitivity analysis using all candidate variables as well as variables selected by LASSO regression. The results showed that the AUC values remained robustly high across both sets, confirming the stability and reliability of model performance.

Based on SHAP analysis, we identified eight key predictors of MetS: waist circumference, blood glucose, CCI, HDL, TyG, systolic blood pressure, age, and CMI. These findings provide reliable information for early screening of MetS. Among these, waist circumference was the most influential predictor, suggesting that central obesity is a stronger predictor of MetS than general obesity.

From a biological perspective, waist circumference is a reliable indicator of visceral fat accumulation (33). Adipose tissue is not merely an energy reservoir; it also functions as an endocrine organ (34). It induces hepatic insulin resistance by releasing free fatty acids and promotes the secretion of inflammatory factors. These effects collectively lead to hyperglycemia, dyslipidemia, and hypertension, ultimately resulting in MetS (35). A large cross−sectional study involving 418,343 working−age adults explicitly stated that waist circumference should be considered a core indicator for the early diagnosis of insulin resistance and MetS (36). Another machine learning study based on 956 adults similarly identified waist circumference as a key predictor of MetS (37).

It is worth noting that Asian populations are more sensitive to the metabolic risks associated with increased waist circumference. A multinational analysis from the UK Biobank indicated that the diabetes risk corresponding to a waist circumference of 88 cm in white women is reached at just 74 cm in Chinese women; similarly, the risk associated with 102 cm in white men is reached at only 88 cm in Chinese men (38). The International Diabetes Federation (IDF) has explicitly recommended lower waist circumference cut−offs for Asian populations (≥90 cm for men and ≥80 cm for women) (39). The Chinese diagnostic criteria for MetS, established by the Diabetes Branch of the Chinese Medical Association and used in this study, differ from the IDF criteria for Asian populations with respect to the waist circumference cutoff for women (85 cm vs. 80 cm, respectively). A large−scale epidemiological survey in China demonstrated that a waist circumference of ≥85 cm in women is useful for diagnosing MetS (40). Adopting the IDF criteria would have included a substantial number of individuals with only mild abdominal obesity, which could have reduced the model’s discriminatory ability. Differences in MetS diagnostic criteria across ethnic groups therefore remain an inherent limitation of this study.

Furthermore, waist circumference measurement is simple, non−invasive, and extremely low−cost, making it particularly suitable for rapid screening in community healthcare settings and thus highly practical for clinical practice.

Skeletal muscle is the largest glucose−utilizing tissue in the body and works in concert with insulin to regulate blood glucose levels (41). Prolonged limb disuse reduces muscle function and

skeletal muscle mass, consequently triggering insulin resistance, disturbed energy metabolic homeostasis, and attenuated adaptability to metabolic stress (42). Abnormally elevated blood glucose levels can induce a chronic low−grade inflammatory state while also adversely affecting glucose and lipid metabolism and vascular endothelial function. These factors collectively contribute to the development of MetS (43).

As an alternative marker of insulin resistance, the TyG index combines fasting triglyceride and glucose levels to provide a more comprehensive assessment of the severity of metabolic disorders (44). Previous studies have shown that an abnormally elevated TyG index is an independent risk factor for MetS (45). Conversely, maintaining a normal TyG index can reduce the risk of various adverse outcomes (46, 47). A cross−sectional study involving 1,560 patients with type 2 diabetes revealed that individuals with a high TyG index (>9.31) had a significantly higher risk of developing MetS compared with those with a normal TyG index (48). The CMI is not only a novel indicator for distinguishing diabetes but also reflects an individual’s obesity and lipid profile, and it is associated with more than ten types of emerging chronic diseases (49, 50). In a study of an ethnic minority group in Southwest China, Chen et al. evaluated the predictive performance of 12 measurable clinical indicators for MetS. The results showed that CMI achieved an AUC ranging from 0.678 to 0.779, ranking second among all indicators, demonstrating that CMI can serve as an independent predictor of MetS (51). As a classic anti-atherogenic lipoprotein, a decrease in HDL level directly reflects an overall disruption in lipid metabolism. Previous studies have shown that higher HDL levels provide a 20% protective effect against the risk of MetS (52). Although advanced age, elevated blood pressure, and an abnormal CCI account for a small proportion of the model and contribute less than the aforementioned predictors, their role as auxiliary predictors cannot be denied.

Finally, a predictive nomogram was constructed by incorporating the predictive factors identified in this study. MetS can be detected at an early stage in both clinical and community−based settings. Targeted interventions can then be implemented for high−risk populations, leading to a more rational allocation of medical and social resources. Furthermore, a structured support system can be established to reduce the incidence of complications, promote muscle strength recovery and functional rehabilitation, and facilitate patients return to their families and society.

## 5 Limitations

This study has several limitations that should be considered. First, the exclusion of relevant psychosocial and environmental factors may limit the comprehensiveness of the diagnostic model. Second, this study lacked independent external testing to validate the model, and the results may not fully reflect its performance across different healthcare institutions. In future studies, we will plan to conduct multicenter external validation to further assess the model’s generalizability. Finally, although this study used the aforementioned predictors to detect MetS, it did not account for potential time−varying effects of these predictors. In future studies, we will strive to integrate these multidimensional, multi−time−point indicators and combine them with independent external validation to construct a more comprehensive and interpretable diagnostic model.

## 6 Conclusion

In this study, we developed and compared seven machine learning algorithms based on data from a clinical retrospective survey to construct a diagnostic model for MetS in patients with DMA. Among these algorithms, the RF model performed best across all evaluation metrics, demonstrating excellent discriminatory ability, stability, and clinical utility. Subsequently, we used SHAP analysis to further identify the key factors associated with MetS, providing a reliable tool for clinical decision−making and a reference for the prevention and intervention of MetS.

## Data Availability

The data analyzed in this study is subject to the following licenses/restrictions: If you have a legitimate request, please contact the corresponding author. Requests to access these datasets should be directed to parida0331@126.com.
